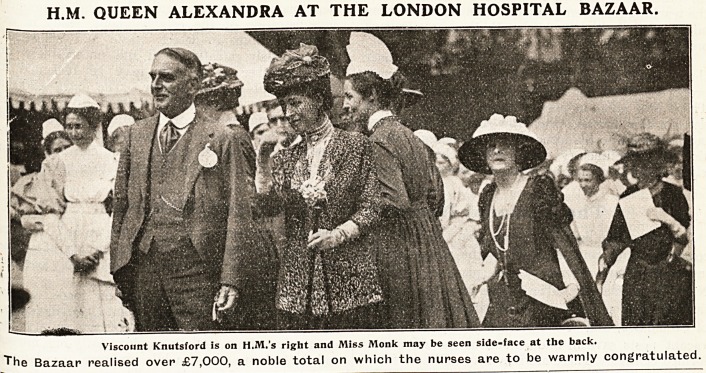# The Sister-Tutor as an Aid to Economy

**Published:** 1921-07-16

**Authors:** 


### THE MATRONS' AND SISTERS' DEPARTMENT.

### H.M. QUEEN ALEXANDRA AT THE LONDON HOSPITAL BAZAAR.

Viscount Knutsford is on H.M.'s right and Miss Monk may be seen side-face at the back.

The Bazaar realised over £7,000, a noble total on which the nurses are to be warmly congratulated.

### THE SISTER-TUTOR AS AN AID TO ECONOMY.

The immediate effect of the Draft Syllabus circulated by the General Nursing Council has been to cause great heart-searchings in all institutions, and particularly in such as are rate-supported. There is very little doubt that the acceptance and loyal carrying out of this Syllabus of training involves additional teaching power in all but the training-schools already highly organised in this respect. It involves in fact the appointment of a special sister, if possible specially prepared for teaching duties, to assume the main burden of instructing probationers in their theoretical studies and in their practical work. At modern rates of salary the sister-tutor cannot be offered less than £100 a year, rising to £150. Including allowances, the extra cost of this to ratepayers or voluntary subscribers must be well over £200. The question which guardians and governors are at present anxiously debating is whether the change will prove to be worth the money.

The modern training-school has come into being very gradually, and in its present form, even in backward institutions, depends entirely for its efficiency and success on the vitality of the superintendent of nurses. Under an energetic, self-controlled, and large-hearted matron almost all is grist which comes to the mill. So long as the probationer be sane in mind and reasonably strong of body she will frame well under a matron skilled in the art of calling forth the good qualities which may be latent, discouraged by early circumstances, yet always there. But the process of training involves a minute knowledge of the probationer's doings in every branch of her work. It entails giving classes, exercising supervision over class work, observation of the extent to which instruction is being assimilated, intimate and first-hand perception of the probationer's character, her weak points, her special talents. It involves also very careful supervision of the manner in which practical work is demonstrated by a large group of ward-sisters, many of them young and inexperienced, some possibly less efficient than could be desired.

At the time when the duties of the matron were first formally defined the training of probationers was a widely different business from what it has lately become. Examinations have altered in character. They are at present a severe test not only of the candidates, but also of those who have prepared them for the ordeal. The outside examiner sits in judgment on more than the nurses. It is not unnatural that the training-school should show symptoms of monopolising more than its fair share of the matron's attention. The other great branch of the matron's duties—the general supervision of the entire establishment, with incessant precautions against waste, and control of every article used in every department under her control, and of each employee—this most necessary side of matronship must inevitably take a second place if the head of the household be encumbered with more teaching duties than can properly be combined with it.

We have had occasion again and again to notice the frequency with which matrons and superintendents of nurses break down before their time, often admittedly from over-pressure of conflicting duties. What causes a breakdown is being overworked, so that always, always, while one thing is being done, another is being neglected. Now when the matron is left single-handed, or with only inefficient helpers, to cope with the modern business of training probationers, as well as with her domestic duties, this position is created. The growth of the teaching has been gradual. One straw has been added after another. At length the camel's back breaks.

But it is not only the matron who suffers under these conditions. The institution suffers in exact proportion to the depletion of her own vital energies. There is a point in every human being at which deterioration begins, when the reserve forces have been explored to the utmost, so that flagging attention and perfunctory routine take the place of vigilant and live superintendence. It may be some time before the effects of this tired-out attitude are observed, but they are inevitably reflected in the expenses. The difference in cost effected by an alert matron, keenly interested in the economical administration of the establishment, ready in expedient, prompt to detect waste and slackness, unconsciously radiating zeal and cheerfulness on every member of the staff, may be reckoned in thousands of pounds a year. The extra £200 a year necessary to free the matron from some of the exacting cares of teaching will be repaid possibly ten times over by setting her free to perform her own special duties, which can never with any hope of success be delegated to others.

## Figures and Tables

**Figure f1:**